# Long-term benzodiazepine prescriptions in community psychiatry clinics

**DOI:** 10.4102/sajpsychiatry.v30i0.2181

**Published:** 2024-05-03

**Authors:** Machipi A. Tau, Mohamed Y.H. Moosa, Fatima Y. Jeenah

**Affiliations:** 1 Department of Psychiatry, Faculty of Health Sciences, University of the Witwatersrand, Johannesburg, South Africa

**Keywords:** long-term, benzodiazepines, prescriptions, community psychiatry, diagnosis, comorbidities

## Abstract

**Background:**

Anecdotal evidence indicates that the prevalence of long-term benzodiazepine prescription is high and not in accordance with accepted prescribing guidelines.

**Aim:**

To determine the prevalence of long-term prescriptions of benzodiazepines and associations thereof in community psychiatry clinics.

**Setting:**

Of the 27 community psychiatry clinics, 5 were randomly selected.

**Methods:**

A descriptive, retrospective, and cross-sectional record review of files of 126 adult patients was conducted, to obtain sociodemographic and clinical characteristics. Descriptive statistics were presented as proportions and percentages. Fisher’s exact test was used to determine any associations between long-term benzodiazepines use and demographic and clinical variables. Regression analyses were performed to determine the significance of any such associations.

**Results:**

Approximately one out of every four patients were prescribed benzodiazepines. Most of the patients were males aged between 18 and 50 years, single and unemployed. The most common psychiatric diagnoses were bipolar disorders and psychotic disorders, and the majority had no comorbid medical illnesses or substance use. Ninety-three per cent of the patients were prescribed long-term (more than 180 days) benzodiazepines. There were no statistically significant associations between prescribing patterns and any sociodemographic and clinical characteristics (*p* > 0.05).

**Conclusion:**

This study found that nearly all the benzodiazepine prescriptions were long-term (over 180 days) and no statistically significant associations between this practice and any sociodemographic and clinical characteristics could be established.

**Contribution:**

There is high prevalence rate of long-term benzodiazepine prescription in community psychiatry clinics, and as such clinical monitoring systems need to be established and enforced.

## Introduction

International treatment and good practice guidelines recommend that before prescribing benzodiazepines to any patient, clinicians should first exclude any physical illness and determine the benefit-to-risk ratio.^1^ The guidelines also recommend that benzodiazepines may be prescribed as a short-term treatment for severe insomnia^2,3^ to counter any side effects of co-prescribed medication^1^ while waiting for the full effect of other treatment modalities to occur^3,4,5^ and to alleviate withdrawal symptoms in patients with substance misuse.^1,6,7^ Benzodiazepines should not be used routinely in patients with anxiety disorders,^2,5,7^ except for symptomatic relief of severe anxiety or panic attacks as they can provide rapid relief. They should be prescribed for only 2 weeks and thereafter reviewed.^1^ The South African Society of Psychiatrists’ treatment guidelines for psychiatric disorders that includes the use of benzodiazepines^8^ are similar to other international treatment guidelines.

Worldwide, variable rates in the prescribing of benzodiazepines have been reported.^9,10,11,12,13,14^ Maust, Blow, and Lin^15^ reported that, in the United States (US), 30.6 million adults (12.6%) used benzodiazepines in 2016, although Agarwal and Landon^16^ reported a slightly lower rate of 7.4% in 2019. The European study of the epidemiology of mental disorders reported a prescribing rate of 18.4% across Europe,^12^ while in South Africa, a study comparing prescribing patterns in Gauteng and the Northern Cape found rates of 7.9% – 8.5% and 9% – 9.5%, respectively.^17^ The variation in published rates by different countries may be attributed, at least in part, to different methodological approaches and clinical settings for example, lower rates may be attributed to possible underreporting by the participants because of stigma, while rates in tertiary outpatient psychiatry clinics may differ from that in community district clinics, because of less specialised staff in community clinics.

Despite not being recommended as first-line treatment by all guidelines,^1,2,4,17^ benzodiazepines in actual clinical practice tend to be prescribed for a broad range of psychiatric conditions, including anxiety, mood, psychotic, personality, and sleep disorders,^10,18,19,20^ and in patients with comorbid medical illnesses.^19,20,21,22^ They are also frequently prescribed for older patients,^23,24,25,26^ females,^27,28,29^ patients with lower education and socioeconomic status,^24^ and those with perceived low levels of support.^24,30^ Although the guidelines recommend that benzodiazepines be prescribed for short periods of 2 weeks initially and then reviewed, several studies have reported a high rate of prescribing benzodiazepines for long-term periods.^18,19,31,32,33^ The World Health Organization (WHO) defined long-term benzodiazepine use as exceeding 180 days,^34^ while others have suggested between 4 and 12 weeks.^33^ The WHO definition is the most frequently used in research and was the definition used in this study.

Inappropriate and long-term prescribing of benzodiazepines by healthcare professionals is one of the leading causes of iatrogenic benzodiazepine use disorders.^31^ The adverse health effects of benzodiazepine use disorder include psychomotor impairment, impairment in judgement and dexterity, forgetfulness, confusion, irritability, aggression, and paradoxical disinhibition.^35^ Tolerance, dependence, and withdrawal symptoms may also occur.^35,36^ Long-term benzodiazepine use may be appropriate in cases such as terminally ill or severely handicapped patients, where the benefits outweigh the risks and side effects.^1,37^

There is a paucity of research in the South African public health sector, especially in district clinics, where anecdotally, long-term benzodiazepine prescribing is a common practice, hence the need for this study. Establishing the prescribing patterns of benzodiazepines at the clinical level may help to detect and prevent long-term use and subsequent adverse health consequences. Such findings would also help district managers develop systems for monitoring benzodiazepine prescribing patterns and ensuring that the recommendations of approved guidelines are correctly implemented.

The study aimed to determine the rate of long-term prescribing of benzodiazepine and to determine any associations between demographic and clinical variables and this prescribing pattern at community psychiatry clinics in Johannesburg health district.

## Research methods and design

The study was a descriptive retrospective and cross-sectional record review. Of the 27 community psychiatry clinics in the Johannesburg health district, five were randomly selected. The names of all 27 clinics were written on pieces of paper and placed in a hat. From the hat, five pieces of paper were randomly drawn, which made up the study sites. The clinics included in the study were Discoverers, Eldorado Park Ext 8, Crosby, Zola, and Hillbrow.

### Study population and sample

The records of all patients aged 18 years and older who attended the community psychiatry clinic and were seen by a doctor from 01 January to 30 June 2019 were eligible for inclusion. The researcher was only available to collect data in the latter half of 2019. The period from 01 January to 30 June 2019 represented the most recent 6-month period before this. The attendees are chronic patients and receive 6-month repeat prescriptions, hence the study period was limited to 6 months to avoid capturing the same patient’s data twice. If the patient had several clinic visits within the study period, only the first visit was considered. Manual randomisation was utilised in that every fifth patient in the register of each of the five community psychiatry clinics was chosen for inclusion in the study. The study sample comprised of 126 patients, 25 from 4 clinics and 26 patients from 1 clinic. All patients below the age of 18 were excluded. The patient records for the sample population were obtained from the clinic registry.

### Data collection and analysis

The relevant information from the clinical notes and prescription sheets was extracted and captured on a Microsoft Excel spreadsheet and analysed using the R (version 3.6.1) statistical software, R Foundation, Free Software Foundation’s GNU project. Vienna Austria. Sociodemographic data included age, gender, marital status, occupational status, and the highest level of education. Clinical data included the primary psychiatric diagnosis, the presence of comorbid medical illness or substance use, the psychotropic medication prescribed, and the type, dose, indication, and duration of the benzodiazepine prescribed.

For meaningful statistical analysis, the categories of some of the variables were collapsed into fewer categories. They included age groups, marital status, occupational status, and the highest level of education attained. Frequencies and percentages were calculated for all variables.

The study population was first divided into two groups, namely: those who were prescribed benzodiazepine and those who were not and were statistically analysed. In a subsequent analysis, the study population was divided into two other groups namely: those who were prescribed long-term benzodiazepines and those who were not. Fisher’s exact test was used to determine any associations between long-term benzodiazepine use and demographic and clinical variables. Multivariate regression analyses were performed to determine the significance of any such associations, if present.

### Ethical considerations

Ethical approval was granted by the University of Witwatersrand Human Research and Ethics and permission to conduct the study in the district by the Johannesburg District Research Committee (Certificate number M190116). Permission to conduct the study in the chosen clinics was obtained from the Johannesburg District Research Committee. No identifying data were captured and each record was given a unique number known only to the researcher as there were no other investigators in this study.

## Results

### Demographic and clinical characteristics of the study population

The study population comprised 126 patients, 25 each from Discoverers, Crosby, Zola, and Hillbrow psychiatric clinics, and 26 from Eldorado Park Ext 8 psychiatric clinic. The demographic and clinical characteristics are presented in Table 1.

**TABLE 1:** Frequency distribution of demographic and clinical characteristics of the study population.

Variables	Study population (*N* = 126)	
	*n*	%
**Gender**		
Male	67	53.2
Female	55	43.7
Unknown	4	3.2
**Age group**		
18–50 years	84	66.7
> 50 years	42	33.3
**Occupational status**		
Employed	9	7.14
Unemployed	66	52.4
Unknown	51	40.5
**Highest level of education achieved**		
Matric or below	59	46.8
Above matric	13	10.3
Unknown	54	42.9
**Marital status**		
Single	85	67.5
Married	23	18.3
Unknown	18	14.3
**History of medical comorbidity**		
Yes	46	36.5
No	80	63.5
**History of substance use**		
Yes	34	27.0
No	92	73.0
**Other psychotropic medications prescribed**		
Yes	106	84.1
No	20	15.9
**Primary psychiatric disorder diagnosis**		
Psychotic	39	31.0
Bipolar	37	29.4
Depressive	21	16.7
Personality	6	4.8
Anxiety	10	7.9
Other	13	10.3

Most of the participants were males (53.2%; *n* = 67), in the age group 18–50 years (66.7%; *n* = 84), single (67.5%; *n* = 85), and unemployed (52.4%; *n* = 66). Long-term benzodiazepine use and adverse side effects are associated with advancing age and hence the age groups were collapsed into two groups, namely 18–50 years and > 50 years. Just below half of the participants had achieved a matric or lower level of education (46.8%; *n* = 59). The most common primary diagnosis was psychotic disorder (31.0%; *n* = 39), followed by bipolar disorder (29.4%; *n* = 37). Personality disorders accounted for only 4.8% (*n* = 6) of the patients. Psychotropic medication was co-prescribed in 84.1% (*n* = 106) of patients. Approximately 63.5% (*n* = 80) of the patients had no comorbid medical illness and 73.0% (*n* = 92) had no comorbid substance use.

Approximately one out of every four patients in the study population was prescribed a benzodiazepine (23.8%; *n* = 30). The demographic and clinical characteristics are listed in Table 2.

**TABLE 2:** Frequency distribution of demographic and clinical characteristics of the group that was prescribed a benzodiazepine and those that were not.

Variables	Benzodiazepine prescribed (*N* = 30)		Benzodiazepine not prescribed (*N* = 96)		OR	CI	*P*
	*n*	%	*n*	%			
**Gender**					1.22	0.48–3.31	0.675
Male	17	56.7	50	52.0	-	-	-
Female	12	40.0	43	44.8	-	-	-
Unknown	1	3.33	3	3.1	-	-	-
**Age group**					0.57	0.23–1.45	0.192
18–50 years	17	56.7	67	70.0	-	-	-
> 50 years	13	43.3	29	30.2	-	-	-
**Occupational status**					0.43	0.01–2.62	0.313
Employed	1	3.33	8	8.33	-	-	-
Unemployed	19	63.3	47	49.0	-	-	-
Unknown	10	33.3	41	42.7	-	-	-
**Highest level of education achieved**					1.70	0.31–17.61	0.719
Matric or below	14	46.7	45	46.9	-	-	-
Above matric	2	6.67	11	11.4	-	-	-
Unknown	14	46.7	40	42.7	-	-	-
**Marital status**					0.76	0.24–2.71	0.585
Single	18	60.0	67	70.0	-	-	-
Married	6	20.0	17	17.7	-	-	-
Unknown	6	20.0	12	12.5	-	-	-
**History of medical comorbidity**					0.46	0.57–3.63	0.392
Yes	13	43.3	33	34.4	-	-	-
No	17	56.7	63	65.6	-	-	-
**History of substance use**					0.61	0.18–1.75	0.358
Yes	6	20.0	28	29.2	-	-	-
No	24	80.0	68	70.8	-	-	-
**Other psychotropic medications prescribed**					1.20	0.37–5.81	0.781
Yes	26	86.7	80	83.3	-	-	-
No	4	13.3	16	16.7	-	-	-
**Primary psychiatric disorder diagnosis**					-	-	-
Psychotic	7	23.3	32	33.3	0.60	0.20–1.07	0.370
Bipolar	10	33.3	27	28.1	1.28	0.47–3.31	0.648
Depressive	4	13.3	17	17.7	0.72	0.16–2.48	0.781
Personality	2	6.67	4	4.17	1.64	0.14–12.12	0.628
Anxiety	2	6.67	8	8.33	0.79	0.08–4.28	1.000
Other	5	16.7	8	8.33	0.22	0.52–8.39	0.229

The most commonly prescribed benzodiazepine was clonazepam (96.7%; *n* = 29), and only one patient (3.3%) was prescribed oxazepam. The mean dose of clonazepam prescribed was 0.8 mg (standard deviation [s.d.= ± 0.61) and the actual doses were 0.25 mg (18.5%; *n* = 5); 0.5 mg (44.4%; *n* = 12); 1 mg (33.3%; *n* = 9); 2 mg (7.4%; *n* = 2), and 3 mg (3.7%; *n* = 1). The dose of oxazepam prescribed was 15 mg. Only three patients (10.0%) had a clear indication recorded for the initiation of a benzodiazepine prescription (two for sedation and one for insomnia). There were no statistically significant differences between this group and the group that was not prescribed benzodiazepines with regard to all demographic and clinical variables (*p* > 0.05) and hence no multivariable analysis could be undertaken.

Twenty-seven (90%) of the 30 patients who were prescribed benzodiazepines met the WHO criterion of over 180 days for long-term benzodiazepine prescriptions (Table 3).

**TABLE 3:** Duration of the benzodiazepine prescription.

Duration	Number of patients	%
1–6 months	2	6.7
6–12 months	1	3.3
> 12 months	27	90.0

Because of the small number of patients not on long-term benzodiazepine use, no meaningful comparisons could be made between the group of patients who were prescribed long-term benzodiazepines and those who were not with respect to the demographic and clinical variables.

## Discussion

Approximately one out of every four patients (23.8%) in the Johannesburg Health District community psychiatric clinics were prescribed a benzodiazepine. While similar high prescribing rates of 18.7% – 24.0% have been reported,^10,32,38^ lower rates of 3.8% – 12.6% have also been reported.^13,14,15^ The variation in reported prescribing rates could be because of differences in the methodology employed, such as study design, settings, and inclusion and exclusion criteria. The high rates in the studies of Ghosh-Nodia and Ahuja^38^ and Haw and Stubbs^11^ may have been as a result of much larger sample sizes, whereas in the studies performed by Haw and Stubbs^11^ and Johnson et al.^32^, the high prescribing rates may have been because the prescription was initiated during hospital admission and continued in the community clinics where the studies were conducted soon after discharge. The high prescribing rates in this study may in part be because of clinicians’ lack of knowledge of benzodiazepine prescribing guidelines,^39^ a lack of capacity and time for the re-evaluation of benefits and risks associated with benzodiazepines,^40^ and failure to provide patient psychoeducation on non-pharmacological interventions to manage symptoms.^15^ In addition, there may have been inadequate monitoring of prescriptions by pharmacists, who are required to serve as gatekeepers.^41^ In South Africa, benzodiazepines are classified as schedule five (S5) drugs by the *Medicines and Related Substances Control Act* No. 101 of 1965, which serves as a control measure to prevent inappropriate prescribing and limit misuse of these drugs.^42^ However, these control measures have not been entirely effective.^21^

Benzodiazepines are divided into four groups based on their elimination half-life. They include ultra-short acting (midazolam, triazolam), short-acting (oxazepam, temazepam), intermediate-acting (alprazolam, bromazepam, lorazepam), and long-acting (clonazepam, diazepam, nitrazepam).^6^ The most frequently prescribed benzodiazepine (96.7%) by the clinicians in this study was clonazepam. Singh and Oosthuizen^18^ also reported that clonazepam was the most prescribed benzodiazepine in their patients, followed by diazepam. However, Summers, Schutte and Summers^43^ reported a preference for the longer-acting benzodiazepines in their public sector study. Generally, in the South African public healthcare sector, clinicians’ choice depends on what is available on the country’s essential medicine list and in the central pharmacy stocks at the time of prescribing.^21,44^ During this study, the benzodiazepines available on the essential medicine list were diazepam, lorazepam, oxazepam, and clonazepam. However, as there are no limitations in the South African private health sector; there is a preference for intermediate-acting benzodiazepines such as alprazolam and bromazepam.^14,29^ Other countries have also shown variation in the choice of benzodiazepine prescribed between the public and private sectors. Lebanon, Ramadaan, Sheik-Taha, and Deep^10^ reported that the most used intermediate-acting benzodiazepines were alprazolam (34.6%) and bromazepam (33.6%). It is unclear what guides and informs the choice of one benzodiazepine over the other in public and private healthcare sectors of different countries. The mean dose of clonazepam was like that of other studies^10,20^ and within the manufacturers’ recommendations.^45^ There was also no progressive increase in the benzodiazepine dosage, as might be expected with the development of tolerance.^46^

This study found that in almost all the patients (90%), the benzodiazepines were prescribed for a long-term period (exceeding 180 days). This rate was considerably higher than the range of 10% – 19% reported in most other studies.^18,32,33^ However, it must be noticed that the Johnson et al.^32^ study had a small sample size, and the benzodiazepines were initiated during admission to a hospital, with only those still on benzodiazepines at community clinics soon after discharge included in their study. The Bernard et al.^18^ review was an observational study at primary healthcare clinics with a larger sample size and included only adult patients with anxiety disorders. Although higher rates of between 60% and 70% have been reported,^12,47^ the Lagnaoui et al.^12^ study included only patients with mood and anxiety disorders, while the Valenstein et al.^47^ study included only depressed patients on antidepressants. Notwithstanding the wide variation in published long-term benzodiazepine prescribing rates, which may be attributed to differing methodologies, the rate found in this study was still high.

It is therefore relevant to determine what factors may be associated with this long-term benzodiazepine prescribing habit. Several studies have reported that long-term prescribing of benzodiazepines has been more common for female rather than male patients.^47,48,49,50^ Females tend to have better help-seeking behaviours than males, which may account for them consulting doctors more often and being on medication for more extended periods.^51^ However, Zandstra et al.^34^ and Sjostedt et al.^24^ reported no gender differences to support this. Contrary to the aforesaid, although not statistically significant, this study found a slight preponderance of males. Franken et al.^52^ also reported most males among their participants. It is likely that this study population, at the outset, consisted of more males. This male preponderance may be attributed to the observation that males tend to have medication non-compliance and poor social reintegration after discharge,^52^ which necessitates regular and ongoing follow-up at community clinics.

This study also found that approximately half of the patients on long-term benzodiazepine use (43.3%) were older than 50 years of age, like findings in the French study of the long-term prevalence of benzodiazepine use.^53^ Patients of advancing age generally have comorbid medical conditions that may warrant benzodiazepine initiation and possible subsequent prolonged use.^54,55^ Chronic medical conditions in the older population have been associated with increasing psychosocial stressors that might precipitate or exacerbate anxiety and depression.^16,56^ However, more than half of the patients in the current sample (56.7%) had no medical comorbidities to support this argument. Gerlarch et al.^56^ reported that low quality of sleep in the elderly is also a predictor for long-term benzodiazepine use. However, this was not a clear indication for prescribing benzodiazepine to the patients in this study. Although this study did not show statistically significant associations between age and long-term prescribing of benzodiazepines, the studies that have demonstrated this have focussed primarily on individuals older than 60 years with chronic diseases with depressive, anxiety, or insomnia disorders.^12,22,28^

Being single with perceived poor social support has also been associated with long-term benzodiazepine use because of an increased likelihood of anxiety and related disorders.^9,30,48,52^ Similarly, a lower socioeconomic status because of a lower level of education and unemployment may add to the anxiety of low social support and perpetuate long-term benzodiazepine use.^9,21,30^ The settings of these studies were similar to our research. Many participants who were prescribed benzodiazepine for long-term use in this study were also either unmarried, divorced, or widowed, had a level of matric or below education, or were unemployed. This may be because they were generally from a relatively poor socioeconomic community with a lower level of education, unemployment, and lower income or disability grants.^53^

The origins of substance use disorders are complex and multifactorial, and there is evidence of a correlation with poor mental health, poverty, low levels of education, unemployment, a lack of social amenities, and social exclusion.^57^ The increasing level of discomfort relating to anxiety may lead to prolonged periods of individuals being prescribed and misusing benzodiazepines. Benzodiazepines may be taken inappropriately to treat the undesirable effects of substance abuse, enhance or augment other drugs’ euphorigenic effects, or induce intoxication.^58^ Several studies have reported that comorbid substance use is also associated with long-term benzodiazepine use.^9,20,22^ A South African study^59^ reported a high prevalence rate of substance use disorders in psychiatric patients; however, no studies reported an association between comorbid substance use and prolonged benzodiazepine use. Our research found that most patients (80%) had no comorbid substance use disorder, similar to Aragaines et al.^53^ This may be because of the patients’ under-reporting, poor documentation by the doctors, or small sample size.

Despite it not being recommended by approved guidelines, common mental disorders such as depression, anxiety, and sleep disorders are often associated with long-term benzodiazepine use.^9,26^ Surprisingly, this study found that most of the patients who were prescribed long-term benzodiazepines had bipolar and psychotic disorders. Similar findings were reported by Johnson et al.^32^ This may be because most of the patients at these clinics have had psychiatric admissions for severe mental illnesses and are down-referred for long-term follow-up.^52^

Only three patients on long-term benzodiazepines had documented reasons for a benzodiazepine being prescribed. It is possible that there were clinical grounds for prescribing a benzodiazepine; however, because of poor record-keeping, the prescribers did not document it. Agarwal and Landon^16^ also reported poor record-keeping. It may be that clinicians fail to record the indications for the use of these drugs because they are not familiar with accepted prescribing guidelines because of limited training or a lack of confidence in correctly prescribing these drugs.^39,60^

### Limitations

There were some limitations to this study. The study population comprised patients from only 5 out of the 27 community psychiatry clinics in the Johannesburg health district, and therefore may not be truly representative. The findings cannot be generalisable possibly because of small sample size and as there is a likely variation in prescribing patterns between clinics and other districts. The relatively small sample size may have limited the ability to detect statistically meaningful differences in the various categories’ analyses. The exceedingly small group of short-term benzodiazepine prescriptions has defaulted this study to merely a description of long-term benzodiazepine prescriptions without any comparisons possible. As in any retrospective study design, some data were missing because of possible poor record keeping. Other reported factors associated with long-term benzodiazepine prescribing were not considered in this study and should be included in the design of future studies.

Notwithstanding the limitations, this study does provide useful insights into the prescribing patterns of benzodiazepines. Its strength lies in the fact that to the best of our knowledge, it is the first study to investigate long-term benzodiazepine use, and the associated sociodemographic and clinical factors in community psychiatric clinics. It also highlights the importance of appropriate and adequate documentation and record-keeping.

### Recommendations

It is recommended that further studies be conducted with larger sample sizes and across the various districts in Gauteng and the rest of South Africa. The studies should also include some of the reported factors associated with long-term benzodiazepine use, but not included in this study.

According to accepted guidelines, clinicians at community clinics should attend regular training on responsible prescribing and reviewing of prescriptions for these drugs. They should be encouraged to psycho-educate patients concerning the advantages and disadvantages of benzodiazepines before initiation and during the process of de-prescribing. District clinical managers and pharmacists should adequately enforce appropriate use of established monitoring systems for continuous reviewing of the prescription of benzodiazepines concerning dosage and duration. Academics and psychiatric societies need to engage with the policymakers in the health department to ensure that there are clear regulatory framework and a regulatory body overseeing benzodiazepine prescription and distribution and that there are monitoring systems to ensure the implementation thereof.

## Conclusion

The high rates of prescribing benzodiazepine and the long duration of prescribing found in this study are of concern. In addition, this study found that there were poor records of the indications for prescribing benzodiazepine. It would imply that the recommended accepted prescribing guidelines are not being adhered to. There is a high risk of developing dependence and the emergence of serious adverse effects especially in the elderly. Apart from impacting the patient’s quality of life, there is a risk of litigation against the Department of Health with financial implications. It demands that the pharmaceutical industry and mental health trainers ensure that regular training on guidelines is carried out and that proper clinical monitoring systems be established.
